# Resolving experimental biases in the interpretation of diffusion experiments with a user-friendly numerical reactive transport approach

**DOI:** 10.1038/s41598-023-42260-5

**Published:** 2023-09-12

**Authors:** Christophe Tournassat, Carl I. Steefel, Patricia M. Fox, Ruth M. Tinnacher

**Affiliations:** 1 Earth and Environmental Sciences Area, Lawrence Berkeley National Laboratory, Berkeley, CA USA; 2 Institut des Sciences de la Terre d’Orléans, Université d’Orléans−CNRS−BRGM, Orléans, France; 3 Department of Chemistry and Biochemistry, California State University East Bay, Hayward, CA USA

**Keywords:** Geochemistry, Nuclear waste

## Abstract

The reactive transport code CrunchClay was used to derive effective diffusion coefficients (*D*_*e*_), clay porosities (*ε*), and adsorption distribution coefficients (*K*_*D*_) from through-diffusion data while considering accurately the influence of unavoidable experimental biases on the estimation of these diffusion parameters. These effects include the presence of filters holding the solid sample in place, the variations in concentration gradients across the diffusion cell due to sampling events, the impact of tubing/dead volumes on the estimation of diffusive fluxes and sample porosity, and the effects of O-ring-filter setups on the delivery of solutions to the clay packing. Doing so, the direct modeling of the measurements of (radio)tracer concentrations in reservoirs is more accurate than that of data converted directly into diffusive fluxes. While the above-mentioned effects have already been described individually in the literature, a consistent modeling approach addressing all these issues at the same time has never been described nor made easily available to the community. A graphical user interface, CrunchEase, was created, which supports the user by automating the creation of input files, the running of simulations, and the extraction and comparison of data and simulation results. While a classical model considering an effective diffusion coefficient, a porosity and a solid/solution distribution coefficient (*D*_*e*_–*ε*–*K*_*D*_) may be implemented in any reactive transport code, the development of CrunchEase makes it easy to apply by experimentalists without a background in reactive transport modeling. CrunchEase makes it also possible to transition more easily from a *D*_*e*_–*ε*–*K*_*D*_ modeling approach to a state-of-the-art process-based understanding modeling approach using the full capabilities of CrunchClay, which include surface complexation modeling and a multi-porosity description of the clay packing with charged diffuse layers.

## Introduction

A characterization of radionuclide diffusion parameters in clayey materials is essential for the performance assessment of natural and engineered barrier systems in radioactive waste storage concepts^1,2^. These parameters include the effective diffusion coefficient (*D*_*e*_), effective porosity (*ε*), and adsorption distribution coefficient (*K*_*D*_), which are characteristic of both clayey materials and the radionuclides under investigation. Values of *D*_*e*_, *ε,* and *K*_*D*_ are empirically determined in the framework of the Fickian diffusion theory. However, these parameters typically become lumped together when a range of physical and chemical processes are present that can be unraveled only by applying multi-scale characterization and modeling techniques^3^. The simplicity of the *D*_*e*_–*ε*–*K*_*D*_ modeling approach, as well as its robustness for cases specific to radioactive waste storage concepts (constant far-field conditions in space and time, trace concentration levels for radionuclides), is in stark contrast to the complexities of the underlying physical and chemical processes. However, the D_e_–ε–K_D_ modeling approach is the current norm in barrier performance evaluation methodology due to its simplicity.

Because the *D*_*e*_–*ε*–*K*_*D*_ modeling approach is empirical in nature, parameter estimation is most often carried out based on the fitting of experimental data. For this purpose, extensive diffusion datasets have been acquired over the past decades. The interpretation of such data relies on solving Fick’s first and second laws of diffusion. Fick’s first law states that the diffusive flux of an aqueous solute (*J* in mol m^−2^ s^−1^) is proportional to its concentration gradient (with *c* in mol m^−3^_water_), and its effective diffusion coefficient *D*_*e*_ (in m^2^ s^−1^)^4^:

1 $$J={-D}_{e}\frac{\partial c}{\partial x}$$

The effective diffusion coefficient can be further expressed as a function of the effective porosity $\varepsilon$ (in m^3^_water_ m^−3^_medium_), a geometrical factor $\tau$ (dimensionless), hereafter referred to as tortuosity, and the self-diffusion coefficient of the aqueous solute ${D}_{0}$ (in m^2^ s^−1^):

2 $${D}_{e}=\varepsilon \tau {D}_{0}$$

Fick’s second law is derived from the mass conservation law:

3 $$\frac{\partial {C}_{tot}}{\partial t}=-\frac{\partial J}{\partial x}$$

where ${C}_{tot}$ is the total concentration of the element of interest in the porous medium (in mol m^−3^_medium_), including aqueous species, surface species, and species in solid phases. The rock capacity factor *α* (in units of m^3^_water_ m^−3^_medium_) relates the total concentration of the (radio)tracer in the porous medium to its aqueous concentration only:

4 $$\alpha =\frac{{C}_{tot}}{c}$$

Rearranging Eq. (4) to $\alpha c={C}_{tot}$, and combining this expression with Eqs. (1) and (3) yields:

5 $$\frac{\partial \alpha c}{\partial t}=\frac{\partial }{\partial x}\left({D}_{e}\frac{\partial c}{\partial x}\right)$$

We will now make two assumptions to relate the rock capacity factor *α* (in m^3^_water_ m^−3^_medium_) to the effective porosity in water-saturated condition *ε* (in m^3^_water_ m^−3^_medium_), the bulk dry density *ρ*_*d*_ (in kg_solid_ m^−3^_medium_) and the adsorption distribution coefficient *K*_*D*_ (in m^3^_water_ kg^−1^_solid_). First, we assume that the tracer is present only in solution and on surfaces (with *C*_*surf*_ in mol kg^−1^_solid_) so that *C*_*tot*_ (in mol m^−3^_medium_) can be expressed as:

6 $${C}_{tot}=c\varepsilon +{C}_{surf}{\rho }_{d}$$

Secondly, we assume that the tracer surface concentration *C*_*surf*_ is linearly related to the aqueous concentration through ${C}_{surf}={cK}_{D}$ (with *K*_*D*_ in m^3^_water_ kg^−1^_solid_). Based on this assumption,

7 $${C}_{tot}=c\varepsilon +{cK}_{D}{\rho }_{d},$$

and based on Eq. (4), *α* can be expressed as:

8 $$\alpha =\varepsilon +{\rho }_{d}{K}_{D}$$

When interpreting diffusion data, the adsorption distribution coefficient is commonly assumed to be representative of an instantaneous and reversible adsorption process. If it is further assumed that the medium is homogeneous, and hence ${D}_{e}$, $\varepsilon$, ${\rho }_{d}$ and ${K}_{D}$ are independent of $x$, then Eq. (5) reduces to:

9 $$\frac{\partial c}{\partial t}=\frac{{D}_{e}}{\left(\varepsilon +{\rho }_{d}{K}_{D}\right)}\frac{{\partial }^{2}c}{{\partial x}^{2}}$$

**Figure 1:**
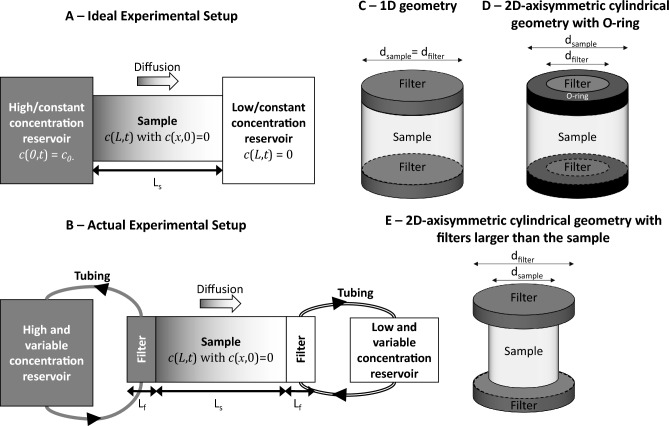
Schematic of an ideal (**A**) and actual (**B**) through-diffusion experimental setup with the goal of achieving constant boundary conditions, i.e., with a constant concentration gradient across the diffusion cell at steady state. (**C**) 1D diffusion geometry. (**D**,**E**) 2D- axisymmetric cylindrical geometries. Tracer cannot diffuse through the plain O-ring of the filter support (**D**), making the diffusional path 2D. The cross-sectional area available for diffusion in the filters is equal to ${\left(\frac{{d}_{filter}}{2}\right)}^{2}/{\left(\frac{{d}_{sample}}{2}\right)}^{2}$ of the cross-sectional area of the sample.

In practice, predictions based on Eq. (9) for a specific set of geometry, initial and boundary conditions are compared to experimental data obtained from a setup representative of the same conditions. Through-diffusion experiments with constant boundary conditions (Fig. 1A), i.e., a constant concentration gradient across the diffusion cell over the course of the experiment, allow for an independent characterization of *D*_*e*_ and *α* in a single experiment. This is accomplished by using the stationary (*D*_*e*_) and transient (*α*) regime of measured diffusive flux data^5^.

Under ideal conditions, the boundary and initial conditions for $c\left(x,t\right)$ in the through-diffusion problem are given by:

10 $$\begin{aligned}&c\left(x,0\right)=0\;{\text{for}}\; 0 < x < Ls\\ &c\left(0,t\right)={c}_{0}\; ({\text{high-concentration reservoir}})\\ & c\left({L}_{s},t\right)=0 \; ({\text{low-concentration reservoir}}) \end{aligned}$$

where *L*_*s*_ is the clay sample thickness in the diffusion cell (m), *c*_0_ (in mol m^−3^) is the aqueous concentration of the element of interest (the tracer, whether radioactive or not) in the high and constant concentration reservoir, and *t* is time (in s). The instantaneous flux *F*_*t*_ (in mol m^−2^ s^−1^) of a diffusive species entering the low-concentration reservoir can then be calculated with an infinite series as a function of time according to^4^:

11 $${F}_{t}=\frac{{c}_{0}\cdot {D}_{e}}{{L}_{s}}+\frac{2\cdot \alpha \cdot {c}_{0}\cdot {L}_{s}}{{\pi }^{2}}\sum_{j=1}^{\infty }\frac{{\left(-1\right)}^{j}}{{j}^{2}}\frac{{D}_{e}\cdot {j}^{2}\cdot {\pi }^{2}}{{L}_{s}^{2}\cdot \alpha }\mathrm{exp}\left(-\frac{{D}_{e}\cdot {j}^{2}\cdot {\pi }^{2}\cdot t}{{L}_{s}^{2}\cdot \alpha }\right)$$

The numerical implementation of Eq. (11) is a truncated version of the infinite series, in which the maximum *j* value can be set high enough to limit the truncation error value to machine precision.

In real experiments (Fig. 1B), the concentration in the high-concentration reservoir is kept as constant as possible by using a large fluid volume. In contrast, the concentration in the low-concentration reservoir is kept as close to zero as analytically/practically possible by replacing the low-concentration reservoir periodically. The experimental tracer diffusive flux (*F*_*exp*_) is then evaluated from the tracer accumulation in the low-concentration reservoir. The cumulated total amount of tracer in the low-concentration reservoir at time ${t}_{n}$_*,*_ $Q({t}_{n})$ (in mol), is obtained with:

12 $$Q({t}_{n})=\sum_{n}{c}_{L}({t}_{n}){V}_{L}({t}_{n})$$

where ${c}_{L}({t}_{n})$ and ${V}_{L}({t}_{n})$ are respectively the tracer concentration (in mol m^−3^), and the volume (in m^3^) of the low-concentration reservoir at sampling time ${t}_{n}$ (in s). The experimental tracer diffusive flux (${F}_{exp}$) is then evaluated by using one of the following backward or central numerical derivative expressions:

13 $${F}_{exp}({t}_{n})=\frac{Q\left({t}_{n}\right)-Q({t}_{n-1})}{\left({t}_{n}-{t}_{n-1}\right)A}$$

or

14 $${F}_{exp}({t}_{n})=\frac{Q\left({t}_{n+1}\right)-Q({t}_{n-1})}{\left({t}_{n+1}-{t}_{n-1}\right)A}$$

where *A* is the cross-sectional area of the sample packed into the diffusion cell (in m^2^). If tubing is used to connect the reservoirs and filters holding the solid sample in place, then *V*_*L*_ also includes the corresponding tubing volumes.

In principle, the least square fitting of *D*_*e*_ and *α* by matching the prediction of *F*_*t*_ based on Eq. (11) with experimental results for ${F}_{exp}({t}_{n})$ calculated from Eq. (13) or (14) is a very convenient way to derive diffusion parameters. The simultaneous fitting of diffusion parameters of non-sorbing tracers for which *K*_*D*_ = 0 and *α* = *ε* (e.g. tritiated water HTO, or ^36^Cl^−^), and sorbing tracers, for which *α* = *ε* + *ρ*_*d*_* K*_*D*_, makes it possible to obtain a comprehensive and operational set of *D*_*e*_*–ε–K*_*D*_ values.

In practice, however, experimental conditions do not often meet the idealized conditions. First, filters at both ends of the sample and tubing connections between the filters and the reservoirs are usually required (Fig. 1B). This complicates the calculations since filters have diffusion properties that differ from those of the solid samples under investigation. Yet, analytical solutions to the diffusion equation can still be obtained under these conditions^6^:

15 $${F}_{t}=\frac{{c}_{0}\cdot {D}_{e}}{{L}_{s}\left(1+\beta k\right)}+2\cdot \alpha \cdot {c}_{0}\cdot {L}_{s}\sum_{j=1}^{\infty }\frac{{D}_{e}\cdot {\omega }_{j}^{2}}{{L}_{s}^{2}\cdot \alpha }\mathrm{exp}\left(-\frac{{D}_{e}\cdot {\omega }_{j}^{2}\cdot t}{{L}_{s}^{2}\cdot \alpha }\right)$$

where ${\omega }_{j}$ is the jth root of the equation (with ${\omega }_{j}\ne 0$):

16 $$a\mathrm{sin}\left({\omega }_{j}\left(1+k\right)\right)+b\mathrm{sin}\left({\omega }_{j}\left(1-k\right)\right)-c\mathrm{sin}\left({\omega }_{j}\right)=0$$

with:

17 $$\begin{aligned} \beta &=\sqrt{\frac{\varepsilon { D}_{e}}{{\varepsilon }_{f}{ D}_{f}}}\\ k&=2 r\frac{{L}_{f}}{{L}_{s}}\\ r & =\sqrt{\frac{{\varepsilon }_{f}{ D}_{e}}{\varepsilon { D}_{f}}}\\ & a=\frac{1}{4}{\left(\beta +1\right)}^{2} \\ b& =\frac{1}{4}{\left(\beta -1\right)}^{2}\\ a&=\frac{1}{2}\left({\beta }^{2}-1\right) \end{aligned}$$

where ${\varepsilon }_{f}$ is the porosity of the filters, ${D}_{f}$ is the effective diffusion coefficient through the filter, and ${L}_{f}$ is the thickness of the filter. Second, the concentration in the high-concentration reservoir can decrease significantly over time in the case of a sorbing tracer. Maintaining a constant concentration level is technically difficult to implement since it requires a very large reservoir volume, potentially a problem since many radiotracers are expensive. Hence, this ideal condition is seldom achieved in experimental practice^7^, and analytical expressions of the diffusive flux must be adapated in conditions where concentrations change significantly in the reservoirs^8^. Third, diffusion equations should, in principle, be solved in 2D- axisymmetric cylindrical coordinates if filters with solid O-rings are used (Fig. 1D,E). Fourth and last, the periodic replacement of low-concentration reservoir solutions generates changes in the concentration gradient across the diffusion cell over the course of the experiment. This further influences the overall diffusive flux as a function of sampling events^9^. No analytical solution has been proposed yet to handle these last two experimental features.

An accurate evaluation of diffusion parameters requires that these experimental biases are specifically taken into account in the fitting procedure, or are mitigated with special experimental procedures^10^. Experimental mitigation strategies include the development of flushed filters in which the reservoir solution is circulated inside of the filters (as opposed to filters at which the solution is circulated at the surface opposite to the sample), thus enabling a more homogeneous solute concentration in the filter and the connected tubing and reservoir^9^. However, all biases and their combinations cannot always be addressed using experimental mitigation strategies or analytical solution development. Thus, ultimately, the estimation of diffusion parameters often relies on approximate analytical solutions^11^ or fully numerical solution strategies^9^. A literature survey reveals that many numerical tools have been developed to interpret diffusion experiments^9,12–14^. However, these tools lack either generality concerning the series of identified biases, easy accessibility by the scientific community, or both.

Reactive transport codes have now been developed for over four decades to model complex interactions in natural systems, while coupling advection, dispersion, diffusion, and sorption reactions^15^. These codes can numerically and iteratively consider an arbitrary number of kinetic or equilibrium reactions together with flow and diffusion equations. Their robustness and accuracy have been tested repeatedly and successfully in benchmark exercises covering a range of applications including diffusion problems^16,17^. Most of these codes are freely available to the scientific community, and in many cases open-source as well^15^. It is thus very surprising that reactive transport models have not been used more extensively to interpret experimental diffusion data. From our own experience, we can identify at least five assumptions that may explain this observation: (1) reactive transport codes are perceived as too difficult to handle by experimentalists; (2) experimentalists and reactive transport modelers are two separate scientific communities with limited communication; (3) reactive transport calculations are too slow to efficiently handle the fitting of diffusion parameters; (4) numerical algorithms in reactive transport codes are not sufficiently accurate compared to analytical solutions; (5) reactive transport codes are not adapted to handle a *D*_*e*_–*ε*–*K*_*D*_ modeling approach because they are focused on process understanding.

With the present contribution, we would like to refute assumptions (3) to (5) that we consider are incorrect and overcome assumptions (1) and (2) by providing the community with a user-friendly, fast, generic, reliable, and free tool, namely the CrunchEase user interface to be used with the CrunchClay code for use interpreting diffusion data with *D*_*e*_–*ε*–*K*_*D*_ conceptual models. We will also show that CrunchEase in conjunction with CrunchClay can be used as a first modeling step that can help to set up a more complex modeling approach that takes into account state-of-the-art theoretical concepts for diffusion and surface processes of contaminants in clayey materials.

## Reactive transport model and investigated systems

### Reactive transport simulator and user interface

Reactive transport calculations of this study were carried out with the code CrunchClay. CrunchClay code capabilities, equations, and the solver scheme have been described in detail in previous publications^15,17–20^.

A graphical user interface, CrunchEase, has been designed to help users build CrunchClay input files and to extract radionuclide (or another tracer) diffusion results. This further allows the readers to re-run the simulations shown in the present study, and to easily use the code to fit their experimental diffusion data. The CrunchEase package is freely available. Installation instructions and snapshots describing the interface are available in the supplementary information. Data and modeling results are plotted in the interface as normalized tracer flux, *F*_*norm*_ (in m s^−1^) as a function of time:

18 $${F}_{norm}({t}_{n})=\frac{{F}_{exp}({t}_{n})}{{c}_{H,init}}$$

where ${c}_{H,init}$ is the initial concentration of tracer in the high-concentration reservoir. In addition, a tabulated text file is produced as an output file that contains modeling results together with corresponding experimental results.

### Model systems

A series of through-diffusion experimental setups with increasing complexity were used to demonstrate the advantages and reliability of reactive transport simulations in a step-wise manner (Table 1). First, experimental setups without filters were modeled with varied *K*_*D*_ values and sample lengths. Second, the same systems were modeled in the presence of filters. Third, sampling events were additionally included. Last, stagnant tubing volumes were added to the simulation as well. For each of these conditions, we characterized how the calculated diffusive fluxes would change by including a particular feature or not. This approach enabled us to quantify the specific influence of each feature on the estimation of diffusion parameters, and thus to quantify the errors induced in the estimation of diffusion parameters by overlooking these features. Where possible, CrunchEase results were compared to analytical solutions to demonstrate the numerical accuracy of our reactive transport modeling approach.

**Table 1:** Summary of input parameters of reactive transport simulations 1–24.

Sim	Sample diameter (mm)	Sample length (mm)	Sample porosity (–)	Sample tortuosity (–)	Filters diameter (mm)	Filters length (mm)	Filters porosity (–)	Filters tortuosity (–)	K_D_ (L kg^−1^)	High reservoir volume (mL)	Low reservoir volume (mL)	High and low reservoir conc	Sampling events	Volume of tubing (mL)	Figure(s)
1	10	10	0.5	0.05	–	–	–	–	0	1000	20	Constant	None	–	3
2	10	5	0.5	0.05	–	–	–	–	0	1000	20	Constant	None	–	3
3	10	10	0.5	0.05	–	–	–	–	1	1000	20	Constant	None	–	3
4	10	5	0.5	0.05	–	–	–	–	1	1000	20	Constant	None	–	3
5	10	10	0.5	0.05	–	–	–	–	2	1000	20	Constant	None	–	3
6	10	5	0.5	0.05	–	–	–	–	2	1000	20	Constant	None	–	3
7	10	10	0.5	0.05	10	1	0.3	0.3	0	1000	20	Constant	None	–	3, 4
8	10	5	0.5	0.05	10	1	0.3	0.3	0	1000	20	Constant	None	–	3, 4, 6
9	10	10	0.5	0.05	10	1	0.3	0.3	1	1000	20	Constant	None	–	3, 4
10	10	5	0.5	0.05	10	1	0.3	0.3	1	1000	20	Constant	None	–	3, 4
11	10	10	0.5	0.05	10	1	0.3	0.3	2	1000	20	Constant	None	–	3, 4
12	10	5	0.5	0.05	10	1	0.3	0.3	2	1000	20	Constant	None	–	3, 4
13	10	10	0.5	0.05	7*	1	0.3	0.3	0	1000	20	Constant	None	–	3, 4, 5
14	10	10	0.5	0.05	7*	1	0.3	0.3	2	1000	20	Constant	None	–	3, 4, 5
15	10	10	0.2	0.025	7*	1	0.3	0.3	0	1000	20	Constant	None	–	3, 4, 5
16	10	10	0.5	0.8	7*	1	0.3	0.3	0	1000	20	Constant	None	–	3, 4, 5
17	10	5	0.5	0.05	10	1	0.3	0.3	0	1000	20	Variable	Every 0.25 day	–	6
18	10	5	0.5	0.05	10	1	0.3	0.3	0	1000	20	Variable	Every 0.5 day	–	6
19	10	5	0.5	0.05	10	1	0.3	0.3	0	1000	20	Variable	Every 1 day	–	6, 7
20	10	5	0.5	0.05	10	1	0.3	0.3	2	1000	20	Variable	Every 0.25 day	–	6
21	10	5	0.5	0.05	10	1	0.3	0.3	2	1000	20	Variable	Every 0.5 day	–	6
22	10	5	0.5	0.05	10	1	0.3	0.3	2	1000	20	Variable	Every 1 day	–	6
23	10	5	0.5	0.05	10	1	0.3	0.3	0	1000	20	Variable	Every 0 to 1 day (random)	–	8
24	10	5	0.5	0.05	10	1	0.3	0.3	0	1000	20	Variable	Every 0 to 1 day (random)	1.5	8

*2D-axisymmetric simulations.

The parameter values chosen were representative of values reported for actual diffusion experiments in the literature^10,21–24^. The self-diffusion coefficient of the tracer was set at *D*_0_ = 2.1 × 10^−9^ m^2^ s^−1^, which corresponds to an effective diffusion coefficient of *D*_*e,sample*_ = 5.25 × 10^−11^ m^2^ s^−1^ [see Eq. (2) and parameters in Table 1]. The sample grain density was *ρ*_*grain*_ = 2840 kg m^3^^25^, which corresponds to a bulk dry density of *ρ*_*d*_ = *ρ*_*grain*_ × (1 − *ε*_*sample*_) = 1420 kg m^−3^.

### Application to real experimental data

As a proof-of-concept, we also used CrunchEase to simulate published through-diffusion data for HTO, Br^−^ and Ca^2+^ in montmorillonite. Raw data (collected volumes and concentrations as a function of time) are available in the published Supplementary Information from Tinnacher et al.^23^, as well as input parameters summarized in Table S1.

## Results and discussion

### Verification of CrunchEase/CrunchClay calculation accuracy

**Figure 2:**
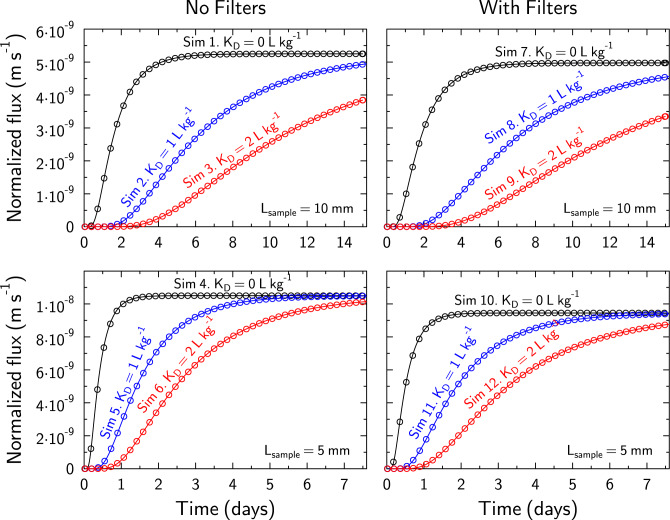
Verification of the accuracy of reactive transport modeling calculation by comparison with analytical equations results for simple model systems, without (left) or with (right) the presence of filters. Simulations 1–12 (Table 1): Comparison of CrunchEase/CrunchClay results (open circles) with analytical (solid lines) Eq. (11) (no filters) or Eq. (15) (with filters) results as a function of sample length (*L*_*sample*_ = 5 mm or 10 mm) and *K*_*D*_ value (from 0 to 2 L kg^−1^).

For simulations 1–6 (Table 1, no filter), diffusion results calculated with CrunchEase/CrunchClay are in almost perfect agreement with the results obtained with analytical solutions, independent of *K*_*D*_ and *L*_*s*_ values (Fig. 2; the graphical user interface CrunchEase makes it possible to visualize this comparison directly).

### The influence of filters

The presence of filters has a marked influence on the outcomes for simulations 7–12 (with filters) compared to simulations 1–6 (no filters) (Fig. 2 and S10), as reported in the literature. The error (Δ*D*_*e*_, Δ*α*) associated with neglecting filter effects can be illustrated by comparing the actual sample diffusion parameters (*D*_*e,ref*_ and *α*_*ref*_), which can be calculated from parameters in Table 1, with the values that can be fitted (*D*_*e,fit*_ and *α*_*fit*_) with Eq. (11) to match the results obtained in the presence of filters (dashed lines on Fig. S10):

19 $$\begin{aligned}\Delta {D}_{e}&=\frac{{D}_{e,ref}-{D}_{e,fit}}{{D}_{e,ref}}\\ \Delta \alpha & =\frac{{\alpha }_{ref}-{\alpha }_{fit}}{{\alpha }_{ref}} \end{aligned}$$

If the presence of filters is neglected in the fitting procedure, effective diffusion coefficients are underestimated and rock capacity factors are overestimated. Furthermore, the influence of filter effects on the absolute *D*_*e*_ value increases with increasing *K*_*D*_ values and *L*_*filter*_/*L*_*sample*_ ratios (Fig. S10 and Table S2), as previously reported^10^. Since it is so easy to include filters in the proposed new modeling package, one should do so as a good standard practice, even when the filters are not expected to affect the results substantially compared to other sources of bias^10^.

### 1D versus 2D axisymmetric cylindrical geometry

**Figure 3:**
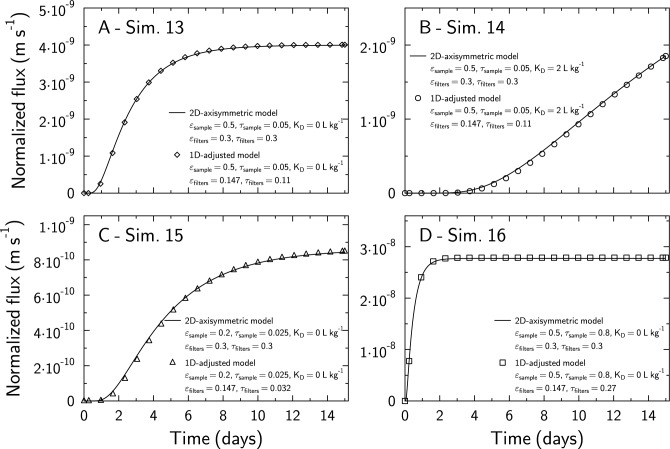
Comparison of 2D axisymmetric cylindrical geometry simulations (plain lines) with 1D geometry simulations (symbols) in which filters porosity was scaled down to 49% of its true value (0.147 instead of 0.3) and its tortuosity was decreased from 0.3 to 0.11 for all 1D simulations. Depending on the contrast of diffusional properties between the sample and the filters, 1D geometry simulations with adapted porosity and tortuosity values for the sample can be in good agreement with the results obtained with 2D axisymmetric cylindrical geometry simulations (**A** sim.13 and 14; **B** sim. 15), or not (**B** sim. 16).

2D- axisymmetric cylindrical simulations 13–16 were carried out with a filter inner diameter (Fig. 1D) of 7 mm, which corresponds to a filter diffusive central sectional area equal to 49% of the total sample cross-sectional area (Table 1; simulations 13–16; Fig. 3). The result of a 1D simulation with a filter porosity scaled down to 49% of its initial value (0.147 instead of 0.3) and a tortuosity value decreased from 0.3 to 0.11 after trial and error iterations proved to be in good agreement with the result of the 2D axisymmetric cylindrical geometry simulation for simulations 13 and 14 (Fig. 3A,B). However, the filter tortuosity value had to be adjusted to other values for simulation 15 and 16 (Fig. 3C,D). If the sample diffusivity is very low compared to that of the filter (Sim. 15, Fig. 3 C), the corrected filter tortuosity should be set at a value far lower than the actual value (0.032 instead of 0.3 in Sim. 15). In contrast, if the sample diffusivity is high compared to that of the filter (Sim. 16, Fig. 3 D), the corrected filter tortuosity should be set to a value similar to that of the actual value (0.27 instead of 0.3 in Sim. 16). In this last case, the lateral diffusion in the sample from the filter diffusive central sectional area is rapid compared to the longitudinal diffusion through the filter and the sample. Thus, the effect of considering a 1D versus 2D axisymmetric cylindrical geometry is almost entirely accounted in the reduction of the porosity that corresponds to a re-scaling of the diffusion sectional area.

The consideration of a 2D axisymmetric cylindrical geometry instead of a 1D geometry (Fig. 1D,E) increases the calculation time significantly. For example, the 1D calculation for simulation 12 (see Table 1) took 23 s, while the equivalent simulation for the 2D axisymmetric cylindrical geometry (Fig. 1D) took 13 min (Intel Core i7, 10th Gen; CrunchClay calculations ran on 4 cores). It may be possible to improve the speed of the 2D axisymmetric cylindrical geometry simulation e.g., by using a coarser grid resolution, or larger time steps. However, hardwired parameters in CrunchEase were chosen to maximize the accuracy of simulations while minimizing users’ concerns in terms of their need to intervene in the parameter optimization process. Instead, the fitting procedure workflow may be greatly accelerated by simulating a representative 1D geometry, before explicitly taking into account the 2D axisymmetric cylindrical geometry, for further model verification and parameter fine-tuning. CrunchEase makes it easy to implement this approach.

### Influence of sampling events

#### Regular sampling time intervals

**Figure 4:**
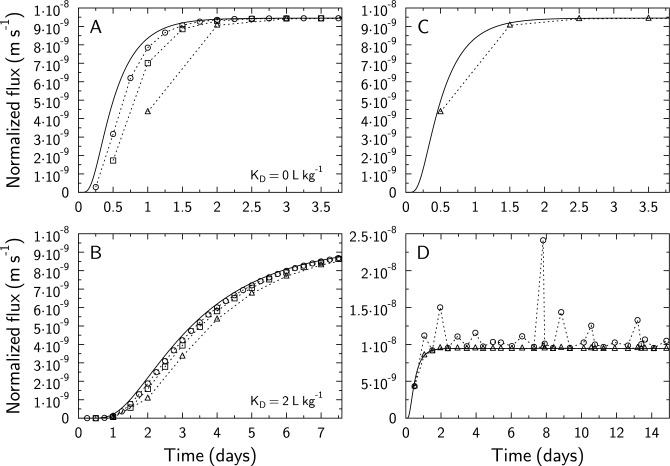
(**A**,**B**) Comparison of diffusion breakthrough curves, computed with CrunchEase/CrunchClay, for different sampling time intervals in simulations 17–19 (**A** *K*_*D*_ = 0 L kg^−1^), and simulations 20 to 22 (**B** *K*_*D*_ = 2 L kg^−1^). Full lines: instantaneous fluxes [calculated with very small time steps, or equivalently with the analytical solution shown in Eq. (15)]. Symbols: fluxes integrated using Eq. (13) over 0.25-day (open circles; simulations 17 and 20), 0.5-day (open squares; simulations 18 and 21) and 1-day (open triangles; simulations 19 and 22) sampling intervals. The dotted lines are eye guides only. (**C**) Instantaneous flux (plain line) and integrated flux with sampling events (triangles; dotted line is an eye-guide) in simulation 19 (1-day sampling interval) with plotting time set at ${t}_{plot}=\left({t}_{n}+{t}_{n-1}\right)/2$. (**D**) Instantaneous flux (full line) and integrated flux (triangles and circles) for a *K*_*D*_ value of 0 and a sample length *L*_*sample*_ = 5 mm, simulated with CrunchEase/CrunchClay. Time intervals were generated randomly. Triangles show results in the absence of tubing volumes (simulation 23), while circles are results for simulations in the presence of tubing (dead) volumes (simulation 24). The dotted lines are eye guides for time-integrated flux only. The plotting time for integrated flux was set at ${t}_{plot}=\left({t}_{n}+{t}_{n-1}\right)/2$.

The periodical replacement of low-concentration reservoir solutions with fresh, tracer-free solutions makes it possible to calculate a diffusive flux based on Eq. (13) or (14). However, this flux calculated at time *t*_*n*_ corresponds to an average (or time integrated) flux from time *t*_*n*−1_ to *t*_*n*_ or *t*_*n*−1_ to *t*_*n*+1_ respectively. If Eq. (13) is used, then time-integrated fluxes are lower than instantaneous fluxes in the early, transient regime of the flux data before diffusion steady-state is achieved (simulations 13–18, Fig. 4A,B). This shifts the breakthrough curves to later times. This bias could be incorrectly interpreted as a retardation factor, and thus to an overestimation of the rock capacity factor. A simple approach for a correction consists in plotting the flux value with ${t}_{plot}=\left({t}_{n}+{t}_{n-1}\right)/2$, which cancels most but not all of the bias (Fig. 4C). As a matter of example, the residual bias in Fig. 4C would correspond to an incorrect estimation of the *K*_*D*_ value at 0.04 L kg^−1^ instead of the actual value at 0 L kg^−1^.

The central difference scheme of Eq. (14) does not produce the same bias as the backward difference scheme of Eq. (13), especially if the sampling time intervals are constant. If they are not constant, then Eq. (14) leads to either a backward or a forward shift in time depending on the position of ${t}_{n}$ with respect to ${t}_{n-1}$ and ${t}_{n+1}$. The resulting uncertainty of the flux values may be higher for the central difference than the backward difference because uncertainties on measured concentrations are propagated over two intervals of time with Eq. (14) instead of one with Eq. (13).

#### Irregular sampling time intervals and influence of tubing volumes

In practice, sampling time intervals vary over the course of a diffusion experiment. These variations have a very significant influence on the time-integrated flux values if tubing volumes are present, which is often the case in through-diffusion experiments (Fig. 4D). First, computed time-integrated fluxes with tubing are always higher than without tubing. This can be explained by the “memory” effect as follows^9^. The solution contained in the tubing volume is not replaced with a fresh, tracer-free solution at the sampling event. This means that this solution provides a significant amount of tracer to the next zero-concentration solution as soon as the new reservoir is connected and circulated. Because the time-integrated flux is calculated based on the accumulation of the tracer in this reservoir and tubing, this initial “flash” increases the tracer concentration results, leading to an overestimation of the flux compared to the real instantaneous flux. Second, variations in sampling time intervals can lead to time-integrated flux variations greater than 100% (Fig. 4D). Again, tubing volume plays a major role here. If a long sampling time interval is followed by a short one, then the tracer concentration increases in the tubing volume proportionally with the length of the time interval. Then, the high-concentration volume contributes significantly more to the total concentration measured in the low-concentration reservoir in the next short sampling time interval. Thus, this leads to an apparent increase in the diffusive flux. This sampling artefact can be corrected by substracting the contribution of the solution in the tubing to the total activity measured in the low-concentration reservoir. However, this correction is not always carried out in published studies. In most cases, no indications about the corrections applied to the activity accumulation and flux calculation are provided.

### Modeling actual experimental diffusion data with CrunchEase

Diffusion data published in the literature sometimes exhibit large variations in diffusive flux calculated at steady-state^26–28^. These variations are most often interpreted in terms of uncertainties, e.g., related to analytical tracer concentration measurements or experimental temperature fluctuations. The present analysis supports previous findings^9^ that a significant fraction of these apparent variations might instead be due to inherent biases resulting from the experimental setups. These biases can be fully taken into account with our reactive transport modeling approach in CrunchEase/CrunchClay, which makes it possible to further decrease the uncertainties related to diffusion parameter estimation. This statement is exemplified with a simulation case based on real experimental data in the next section.

Through-diffusion data are most often published (plotted) in the form of flux and/or total diffused concentrations as a function of time. Unfortunately, none of these representations convey the original raw data in terms of tracer concentrations and exact volumes of reservoir solutions and tubings.

**Figure 5:**
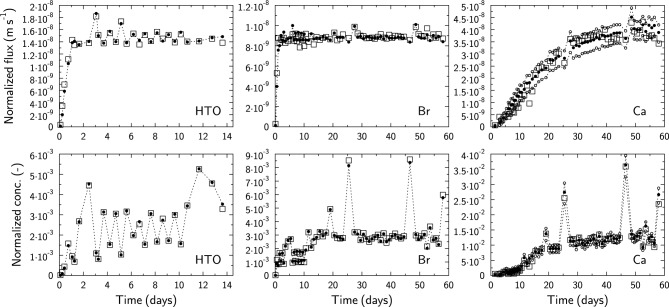
Modeling with CrunchEase/CrunchClay of HTO, Br, and Ca diffusion data from Tinnacher et al.^23^. Open squares: experimental data. Closed and open circles: modeling results. HTO model parameters: *τ*_*clay*_ = 0.049; *ε*_*clay*_ = 0.72. Br model parameters: *τ*_*clay*_ = 0.04; *ε*_*clay*_ = 0.55. Ca model parameters: *τ*_*clay*_ = 1.1–2.3; *ε*_*clay*_ = 0.72; *K*_*D*_ = 79 L kg^−1^. Closed circles: model with *τ*_*clay*_ = 1.6; Open circles: model with *τ*_*clay*_ = 2.3 (upper curve) or *τ*_*clay*_ = 1.1 (lower curve). The dotted lines are eye guides for modeling results only. Other (fixed) parameters are given in Table S1 in the supplementary materials.

However, Tinnacher et al.^23^ have provided original raw and tabulated data, which can be used as described in the following. Experimental data and modeling results are plotted as normalized flux as a function of time, as well as low-concentration reservoir concentrations as a function of time (Fig. 5). We believe that the latter representation is preferred for evaluation of the agreement between experimental and modeling results because it does not include any propagated error from the data interpretation and the consideration of two measurements at time *t*_*n*−1_ and *t*_*n*_ or *t*_*n*+1_. The former representation was included nonetheless to allow for comparisons with previously published data.

The experiment was modeled with a 1D geometry using the same dimensions as those provided by Tinnacher et al.^23^. HTO data were modeled by adjusting only the tortuosity of the clay materials (*τ*_*clay,HTO*_ = 0.049, corresponding to *D*_*e,HTO*_ = 7.51 × 10^−11^ m^2^ s^−1^), other parameters (sample porosity *ε*_*clay,HTO*_ = 0.72, filter porosity *ε*_*filter,HTO*_ = 0.25, and filter tortuosity *τ*_*filter,HTO*_ = 0.43) having been determined independently from the diffusion experiments^23^.

Modeling results, which almost perfectly match the measured concentrations (Fig. 5), clearly demonstrate the power of the proposed reactive transport modeling approach in terms of reproducing the effects of biases that are unavoidable due to the experimental setup, including the presence of filters, tubing ‘dead’ volumes, and variable duration sampling events. The same was true for experimental Br^−^ diffusion data for which two parameters were fitted: *τ*_*clay,Br*_ = 0.040; *ε*_*clay,Br*_ = 0.55, corresponding to *D*_*e,Br*_ = 4.4 × 10^−11^ m^2^ s^−1^ and *α*_*Br*_ = 0.55 (Figs. 5), as well as for Ca^2+^ diffusion data for which three parameters were fitted: *τ*_*clay,Ca*_ = 1.1–2.3 (Figs. 5 shows that it is not possible to give a better estimated than this range of values); *ε*_*clay,Ca*_ = 0.72; and *K*_*D,Ca*_ = 79 L kg^−1^ , corresponding to *D*_*e,Ca*_ = 6.28 × 10^−10^ m^2^ s^−1^ to 1.26 × 10^−9^ m^2^ s^−1^ and *α*_*Ca*_ = 63.5 (Fig. 5). The *τ*_*clay,Ca*_ value fitted here differs significantly from the value reported in Tinnacher et al.^23^. The difference is due to improper handling of filter tortuosity in PHREEQC calculations carried out by Tinnacher et al.^23^ for the Ca^2+^ diffusion simulation.

### Beyond the *D*_*e*_*–ε–K*_*D*_ modeling approach

Although CrunchEase is not (yet) intended to handle the creation of CrunchClay files with complex solution chemistry and porosity distributions, the code can be used to create input files with a specific geometry of interest (1D or 2D rotational, presence or absence of filters, tubings, actual reservoirs volumes, etc*.*), and can also handle sampling events of the low-concentration reservoir. These files (text files with free format) can then be modified directly by the user to include additional chemical reactions and porosity characteristics, such as surface charge and the diffuse layer pore distribution of the clay (a model input file is provided in the Supplementary Materials as a template for interested users).

**Figure 6:**
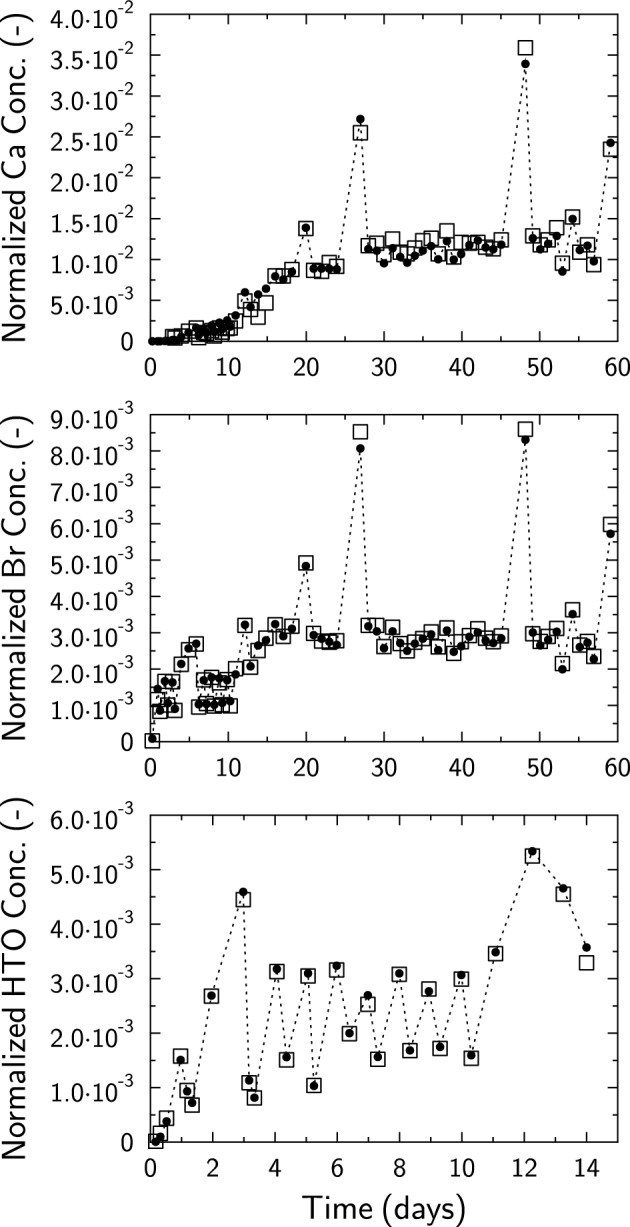
Dual porosity (bulk and diffuse layer) model (black circles; dotted lines are eye guidelines) applied to diffusion data (open squares) from Tinnacher et al.^23^. Model input files were modified from files built by CrunchEase and used to simulate and plot Fig. 5. Ca^2+^, Br^−^_,_ and HTO diffusion were modeled simultaneously. Ca^2+^ and Br^−^ input files were the same because of identical sampling times (same experiment), but the HTO input file was handled separately (different experiment, sampling times, and sampled volumes). Modeling parameters were otherwise the same (see text).

The strength of this approach is illustrated with a dual-porosity model to simulate Ca^2+^, Br^−^ and HTO diffusion data from Tinnacher et al.^23^ (Fig. 6). Model input files were modified from the input files built by CrunchEase and used to simulate and plot Fig. 5. The porosity of the clay sample was split into two contributions: a bulk porosity (*ε*_*bulk*_) and a diffuse layer porosity (*ε*_*DL*_) in which the surface charge (0.9 mol kg^−1^_clay_) of montmorillonite was compensated (see^18,23,29–31^ for full details about theory and parameters). An almost perfect data fit was obtained with the following parameters (Fig. 6): *ε*_*bulk*_ = 0.19; *ε*_*DL*_ = 0.53; *τ*_*bulk*_ = 0.1; *τ*_*DL*_ = 0.032; log *K*_*Na*_ = 0 for the surface complexation reaction:

20 $$> {\text{Surf}}^{ - } + {\text{Na}}^{ + } \rightleftharpoons \, > {\text{SurfNa}}$$

and log *K*_*Ca*_ = 0.7 for the surface complexation reaction:

21 $$2 > {\text{Surf}}^{ - } + {\text{Ca}}^{2 + } \rightleftharpoons \, > {\text{Surf}}_{2} {\text{Ca}}$$

While the model prediction using a dual-porosity model (Fig. 6) is not better than the model prediction made with a simple *D*_*e*_*–ε–K*_*D*_ modeling approach (Figs. 5), it has the advantage to produce model parameters that are more realistic with regards to sample porosity properties. In this respect, CrunchEase can help simplify the use of CrunchClay to achieve process level understanding.

## Conclusions

We have demonstrated that a reactive transport modeling approach that includes the full geometrical and operational complexity of an experiment, such as heterogeneities and time-dependent sampling effects is a very powerful tool for the interpretation of diffusion data. While a classical *D*_*e*_–*ε*–*K*_*D*_ model may be implemented in any reactive transport code, the development of the new graphical user interface CrunchEase makes it easy to apply by experimentalists without background in reactive transport modeling. CrunchEase can also be used to fit through-diffusion data from experiments with changing boundary conditions (no reservoir replacement; not shown in this paper). In the future, the code may be easily extended to other types of diffusion experiments, such as in-diffusion experiments.

Since experimental biases can be modeled explicitly, the accuracy of calculated diffusion parameters with a reactive transport approach is expected to be better than for the application of analytical solutions, which cannot include the full complexities of real experiments. The numerical efficiency of CrunchClay makes our approach suitable to fit model parameters very quickly. For instance, for 1D problems simulating a diffusion experiment with an experimental time frame of approximately 50 days, the model running time is most often less than 1 min. This approach could also be used easily with parameter estimation softwares such as PEST^32^.

On a final note, CrunchEase makes it easier to transition from a *D*_*e*_–*ε*–*K*_*D*_ modeling approach to a reactive transport modeling approach taking into account surface-enhanced diffusion processes and advanced chemical reactivity models such as surface complexation models. Very few reactive transport codes can handle all of these features^31^, which currently makes the CrunchClay-CrunchEase combination unique. This will hopefully help to facilitate the dialog between experimentalists, modelers, and radioactive waste agency decision-makers regarding the most recent concepts applied to diffusion in clayey materials. This may ultimately lead to an improved decision-making process by radioactive waste agencies.

### Supplementary Information

Supplementary Information.

## Data Availability

The CrunchEase interface and data are available on an open Github repository: https://github.com/Tournassat/CrunchEaseForAll/releases/tag/v1.0.0.
