# Supplementary material for: Resolving experimental biases in the interpretation of diffusion experiments with a user-friendly numerical reactive transport approach
Source: Sci Rep. 2023 Sep 12;13:15029. doi: 10.1038/s41598-023-42260-5 (PMC10497684; doi:10.1038/s41598-023-42260-5)
Supplement: Supplementary file 1 — Supplementary Information. [file 41598_2023_42260_MOESM1_ESM.docx]

**Supplementary Information**

**Resolving Experimental Biases in the Interpretation of Diffusion Experiments with a User-Friendly Numerical Reactive Transport Approach**

Christophe Tournassat^1,2^, Carl Steefel^1^_,_ Patricia Fox^1^, Ruth Tinnacher^3^

**Affiliations:**

^1^ Earth and Environmental Sciences Area, Lawrence Berkeley National Laboratory, Berkeley, CA, USA

^2^ Institut des Sciences de la Terre d’Orléans, Université d’Orléans−CNRS−BRGM, Orléans, France

^3^ Department of Chemistry and Biochemistry, California State University East Bay, Hayward, CA, USA

**Corresponding author:** [ctournassat@lbl.gov](mailto:ctournassat@lbl.gov) or [christophe.tournassat@univ-orleans.fr](mailto:christophe.tournassat@univ-orleans.fr)

CONTENT

[1. CrunchEase Quick Tutorial 2](#_Toc129074973)

[1.1. Installation (for Windows users) 2](#_Toc129074974)

[1.2. Run a simple diffusion calculation 2](#_Toc129074975)

[1.3. Compare simulation results with experimental measurements 5](#_Toc129074976)

[1.4. Adding complexity to your CrunchClay simulation 6](#_Toc129074977)

[2. Experimental data from Tinnacher et al. (2016) 7](#_Toc129074978)

[3. Influence of the presence of filters on estimations of *D_e_*, and *α* 8](#_Toc129074979)

[4. References 9](#_Toc129074980)

## CrunchEase Quick Tutorial

### Installation (for Windows users)

Download the file *CrunchEase_Setup.exe* from: <https://github.com/Tournassat/CrunchEaseForAll/releases/tag/v1.0.0>.

Double-click on *CrunchEase_Setup.exe* to install CrunchEase in the directory *C:\Users\* ***YourUserName*** *\Documents\CrunchEase* (follow the instructions and accept the installation). At the moment, you cannot change the install directory.

Go to *C:\Users\****YourUserName****\Documents\CrunchEase*.

Double-click on *w_ifort_runtime_p_2022.2.0.3790.exe* to install the external Intel libraries that are necessary to run CrunchClay.

Reboot your computer.

Go to *C:\Users\* ***YourUserName*** *\Documents\CrunchEase*.

Your directory should contain at least the items shown in Figure S1.


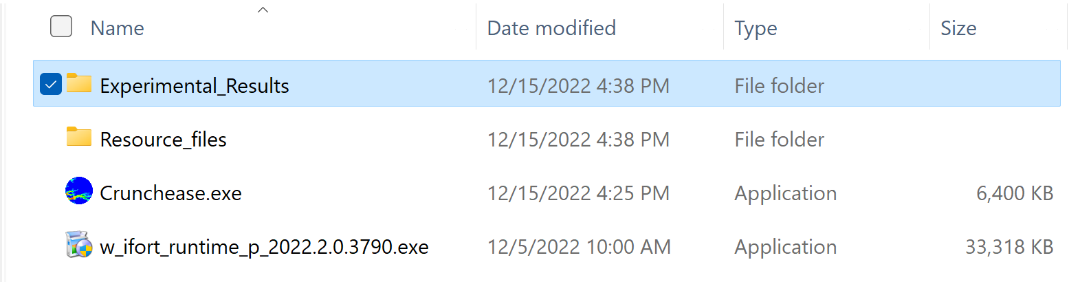


Figure S1. Screenshot of the CrunchEase install directory.

### Run a simple diffusion calculation

Double-click on *CrunchEase.exe*.

Figure S2. Screenshot of the main window of the CrunchEase interface.

Click on “**Model your Diffusion Experiment With CrunchClay**” (Figure S2).

Figure S3. Screenshot of the Diffusion window of the CrunchEase interface.

Click on “**1D Tracer Through-Diffusion**” (Figure S3).

You are ready to run your first simulation with CrunchEase/CrunchClay (Figure S4).

Figure S4. Screenshot of the 1D- Through-Diffusion window of the CrunchEase interface. Follow numbered instructions to run your first simulation.

After a short time, you obtain your results (Figure S5).

Figure S5. Screenshot of the 1D- Through-Diffusion window of the CrunchEase interface after calculation.

Figure S6. Screenshot of the Tutorial directory after calculation.

Open the newly created directory, which is named after your Experiment Title (Figure S6).

Figure S7. Screenshot of the simulation directory (named after your Experiment Title) after calculation.

You will find in the new directory a text file *GraphFile.dat* (Figure S7) that you can use to plot your simulation results with the plotting software of your choice.

Simulation conditions and parameters are saved in the file *SimulationConditions.save*, which is used by CrunchEase to reload saved parameters from an existing simulation name (in the box Experiment Title).

### Compare simulation results with experimental measurements

It is always possible to compare the computed flux with your data using the output file *GraphFile.dat*. However, CrunchEase offers the possibility to directly compare tracer concentrations measured in the downstream reservoir with simulation results. This capability enables better characterization of diffusion parameters while considering experimental setup characteristics more accurately (see the text of the main paper for further explanations). The practical procedure is explained below.

Create a new simulation with a new name, *e.g.* TestWithData.

In the field Exp. Data File, Select a data file (take Ruth_Br.txt for example) in the directory.

**If you want to create a new data file, it must be saved in the *Experimental_results* directory**, so that CrunchEase can find it.

Experimental data files have a simple structure:

Elapsed Time (day) Br rel. Conc Vol low C res (mL)

0.25 2.22E-05 20.15

0.93 1.16E-03 19.7

1.23 8.37E-04 19.99

1.86 1.66E-03 20.03

2.23 1.01E-03 20.05

The first line is a header line that is not used by CrunchEase. Then, starting from the second line, data values must be entered on the same line for each sampling time:

- the elapsed time (in days),
- the tracer relative concentration in the downstream reservoir (dimensionless),
- the volume of the downstream reservoir (in mL).

The three values may be separated with spaces, tabulations, commas, or semicolons.

Tracer relative concentration corresponds to the actual concentration divided by the initial concentration in the upstream (high concentration) reservoir.

Figure S8 shows the options and parameters selected to model Br diffusion data from Tinnacher et al.


(2016).


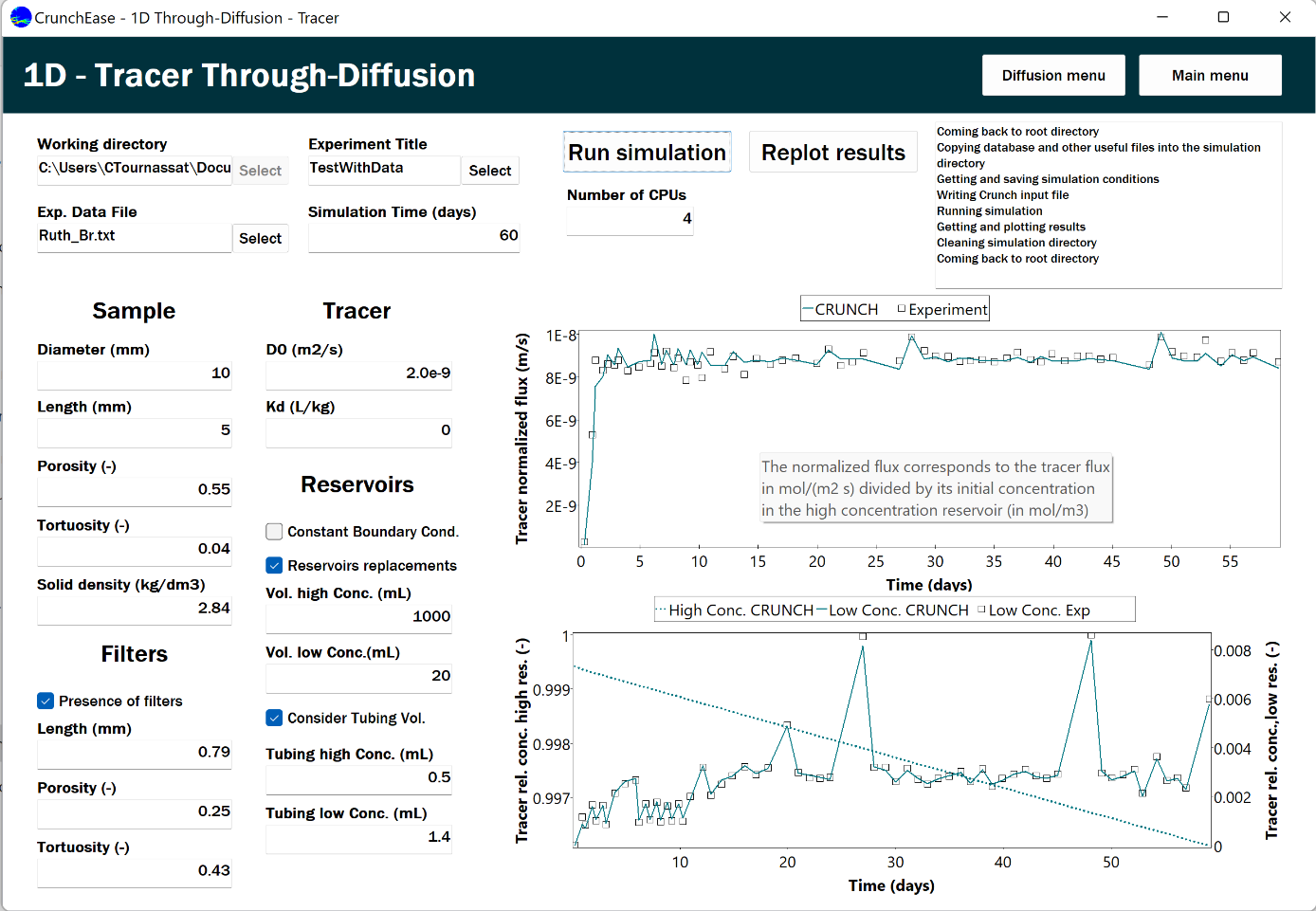


Figure S8. Screenshot of the 1D- Through-Diffusion window of the CrunchEase interface with options and parameters selected to model Br diffusion data from Tinnacher et al.


(2016).

### Adding complexity to your CrunchClay simulation

The directory contains all files (Figure S9) that are necessary to re-run the simulation with or without using CrunchEase, *i.e.* using directly CrunchClay. Running *CrunchFile.in* with CrunchClay will start the entire calculation. Files with the *_resX.in* termination (X is an integer) are restart files used by CrunchClay at each sampling event. These files must not be changed.

The chemical system considered in the simulation can be made more complex by changing chemical inputs in *CrunchFile.in*. These changes necessitate additional knowledge of CrunchClay, which is not covered in this tutorial. In the future, the interface will be enriched with additional capabilities that will help the users to learn and take full advantage of Crunch codes capabilities.

Figure S9. Screenshot of the TestWithData simulation directory after CrunchEase calculation.

## Experimental data from Tinnacher et al. (2016)

Table S1. Summary of input parameters of reactive transport simulations used to model diffusion data from Tinnacher et al.


(2016).

|  | Sample | Filters* |
| --- | --- | --- |
| Diameter | 10 mm | |
| Length | 5 mm | 0.79 mm |
| Porosity | Fitted value | 0.25 |
| Tortuosity | Fitted value | 0.43 |
|  | Tubing high-conc. | Tubing low-conc. |
| Volumes | 0.5 mL | 1.4 mL |
|  | Diffusion coefficients | |
| HTO | 2.13×10^-9^ m^2^ s^-1^ | |
| Br^-^ | 2.00×10^-9^ m^2^ s^-1^ | |
| Ca^2+^ | 7.93×10^-10^ m^2^ s^-1^ | |

*from


(Molera, 2002; Molera et al., 2003; Tinnacher et al., 2016)

## Influence of the presence of filters on estimations of *D_e_*, and *α*

Table S2. Influence of neglecting filter effects on estimations of *D_e_*, and *α* in simulations 7 to 12 (see Table 1).

|  | *K_D_* = 0 (L kg^-1^) | *K_D_* = 1 (L kg^-1^) | *K_D_* = 2 (L kg^-1^) |
| --- | --- | --- | --- |
| *L_sample_* = 10 mm, *L_filter_* = 1 mm | | | |
| $\Delta D_{e}$ (%) | -4.7 | -4.7 | -4.7 |
| $\Delta\alpha$(%) | +10 | +6.7 | +5.9 |
| *L_sample_* = 5 mm, *L_filter_* = 1 mm | | | |
| $\Delta D_{e}$ (%) | -10 | -10 | -10 |
| $\Delta\alpha$(%) | +12 | +9.8 | +9.2 |

Figure S10. Simulations 1 to 12. Comparison of diffusion breakthrough curves calculated in CrunchEase/CrunchClay in the absence (full lines) and presence (dashed lines) of filters, as a function of sample length (*L_sample_* = 5 mm vs. 10 mm) and *K_D_* value (from 0 to 2 L kg^-1^).

## References

Molera, M., 2002. On the sorption and diffusion of radionuclides in Bentonite Clay.

Molera, M., Eriksen, T., Jansson, M., 2003. Anion diffusion pathways in bentonite clay compacted to different dry densities. Appl. Clay Sci. 23, 69–76.

Tinnacher, R.M., Holmboe, M., Tournassat, C., Bourg, I.C., Davis, J.A., 2016. Ion adsorption and diffusion in smectite: molecular, pore, and continuum scale views. Geochim. Cosmochim. Acta 177, 130–149.
